# Three-decade long fertilization-induced soil organic carbon sequestration depends on edaphic characteristics in six typical croplands

**DOI:** 10.1038/srep30350

**Published:** 2016-08-05

**Authors:** Feng Liang, Jianwei Li, Xueyun Yang, Shaomin Huang, Zejiang Cai, Hongjun Gao, Junyong Ma, Xian Cui, Minggang Xu

**Affiliations:** 1 National Engineering Laboratory for Improving Quality of Arable Land, Institute of Agricultural Resources and Regional Planning, Chinese Academy of Agricultural Sciences, Beijing, 100081, China; 2 College of Resource and Environment, Northwest Sci-Tech University of Agriculture and Forestry, Yangling, Shaanxi 712100, China; 3 Institute of Plant Nutrition and Agricultural Resources, Henan Academy of Agricultural Sciences, Zhengzhou, Henan 450002, China; 4 Institute of Agricultural Resources and Environment, Jilin Academy of Agricultural Sciences, Changchun, Jilin, 130033, China; 5 Institute of Upland Agriculture, Hebei Academy of Agricultural Sciences, Hengshui, Hebei, 053001, China; 6 Heihe Academy of Agricultural Sciences, Heihe, Heilongjiang, 164301, China

## Abstract

Fertilizations affect soil organic carbon (SOC) content but the relative influences of the edaphic and climate factors on SOC storage are rarely studied across wide spatiotemporal scales. This study synthesized long-term datasets of fertilization experiments in six typical Chinese croplands, and calculated annual C input from crops and manure amendments, changes in SOC storage (ΔSOC) and C sequestration efficiency (i.e. the percentage of soil C change per unit of C input, hereafter referred as CSE) in 0–20 cm soil over three decades. Three fertilization treatments include no fertilization (CK), chemical nitrogen, phosphorus and potassium fertilizers (NPK) and combined chemical fertilizers and manure (NPKM). Results showed significant fertilization effects on C input and ΔSOC (NPKM>NPK>CK), and significantly higher CSE in Qiyang at Hunan than Zhengzhou at Henan and Heihe at Heilongjiang. The variance partitioning analysis (VPA) showed more variance of CSE can be explained by edaphic factors (up to 39.7%) than other factors. Furthermore, soil available N content and pH were identified as the major soil properties explaining CSE variance. This study demonstrated key controls of soil fertility factors on SOC sequestration and informs the need to develop strategic soil management plan to promote soil carbon sequestration under long-term intensive fertilization.

Soil organic carbon (SOC) is the largest C pool of terrestrial biosphere, more than twice that of the C stored in vegetation biomass and in theatmosphere^1^,^2^. Soil organic matter is a key component of agricultural soil and its content largely determines soil fertility, productivity and sustainability of arable lands^3^,^4^,^5^. SOC changes will exert strong feedbacks on global climate change^6^,^7^. About 10% of organic carbon reserves (140~170 Pg, 1 Pg = 10^15^g) in farmland ecosystem is the most active part of world’s terrestrial soil carbon pool. Chinese farmland harbors organic carbon reserves of about 25–27 Pg and plays an important role in the global carbon budget^8^,^9^. Increase of SOC in croplands has been considered as one of the win-win strategies as it can enhance food security and potentially mitigate global climate change^10^,^11^,^12^,^13^. Therefore, how to maintain and even improve SOC content in cropland has become a pressing issue of modern soil science for the sake of farmland quality, food safety and global climate change.

SOC storage depends on the balance of organic carbon inputs and outputs^14^,^15^,^16^. The SOC change is a dynamic process and the most direct and effective method to increase SOC is organic matter input via plant residue, root deposits and exudates, and manure amendments^14^,^15^,^17^,^18^,^19^. The amount of these organic C input incorporated into SOC pool however were regulated by not only the amount of C input but also the conversion rate of C input, which is hereafter named carbon sequestration efficiency (i.e. CSE) in the current study. CSE denotes C stock change per unit C added^20^. CSE of exogenous organic carbon reflects an integrated measure of soil carbon storage changes under different conditions over a long time period and is important in scientific research and for production practice^21^,^22^. The use of CSE is particularly advantageous for multiple comparisons between sites and treatments.

SOC changes are affected significantly by climate, organic C input, and edaphic factor. In general, higher temperature and more precipitation may produce a favorable soil environment for soil microbial communities, resulting in rapid mineralization of OC input and a lower CSE^23^,^24^,^25^,^26^. Although climate trends could account for 10% of the stagnation in European wheat and barley yields^27^, climate factors only account for 0.14% of variance in mass remaining in a straw decomposition experiment^28^. On the other hand, CSE could vary significantly between animal manure and crop residues on a global scale, but is directly proportional to the amount of carbon input^29^,^30^. Nevertheless, the soil physiochemical properties are usually regarded closely relevant with SOC transformations such as soil texture, pH and so on^19^,^31^,^32^. Last, CSE is also a function of time^12^,^28^,^33^. Even though, prediction of SOC changes is however difficult due to a suite of complex and high-order interactions of these multiple factors. The relative importance of each of these factors on SOC change has been rarely studied particularly based on long-term fertilization experiments.

Across highly diverse climatic zones, management practices and soil types in China, dozens of fertilization experiments have been established since 1970’s. A large volume of high-frequency datasets in soil physiochemical properties, climate record and farm manure amendments have been accumulated^28^,^34^,^35^. Besides no fertilization treatment, most of these established Chinese fertilization experiments can be primarily categorized into two groups: chemical fertilizers alone or combined with manure input^35^,^36^. This experiment design enabled one to explore the relationship between SOC change and C input over a large gradient of organic C input across a wide range of spatiotemporal scales. However, previous studies usually focused on the short-term SOM humification coefficient analysis based on a single or a few experimental sites, lacking of long-term comparative studies on SOC transformation. The relative importance of each of multiple factors on SOC change remained rarely explored.

Given the three-decade long continuous record of climate, chemical and organic fertilizer input, and soil physiochemical properties, the well-maintained Chinese national long-term fertilization network allowed one to study the importance of overall and individual factors on SOC change and its relationship with C input. In this study, fertilization experiments at six typical Chinese cropland soils were selected to examine the temporal pattern of C input, ΔSOC and CSE across different sites; explore the main and interactive effects of fertilization treatment and site on C input, ΔSOC and CSE; and elucidate the relative importance of climatic, management and edaphic factors on CSE. By identifying the specific key controls of soil fertility factors on soil carbon sequestration potentials, this study is expected to promote fertilization and management efficiency in typical upland soils in China or other countries.

## Results

### C input to soil under long-term fertilization

**Figure 1:**
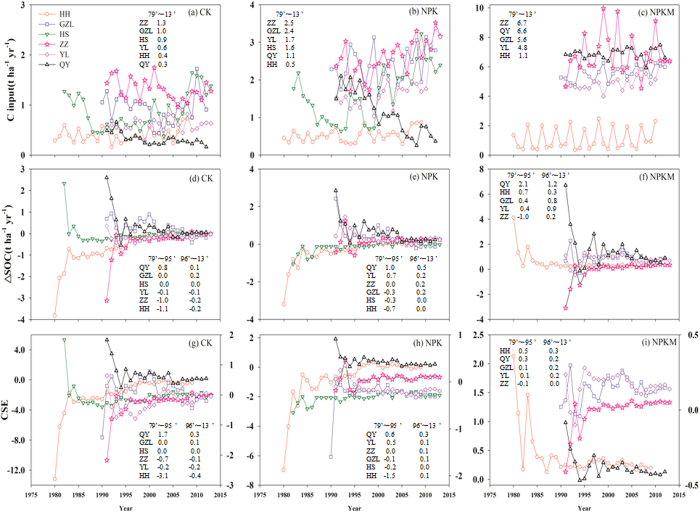
The carbon input (t ha^−1^ yr^−1^), change in SOC storage (ΔSOC, t ha^−1^ yr^−1^) and carbon sequestration efficiency (CSE) from the initial year to 2012 under CK, NPK and NPKM treatments at six fertilization experimental sites in China (panels a~i). The inset in each panel denotes the average carbon input, ΔSOC or CSE during 1979 to 1995 and during 1996 to 2012 at each specific site displayed in a descending order from top to bottom.

**Figure 2:**
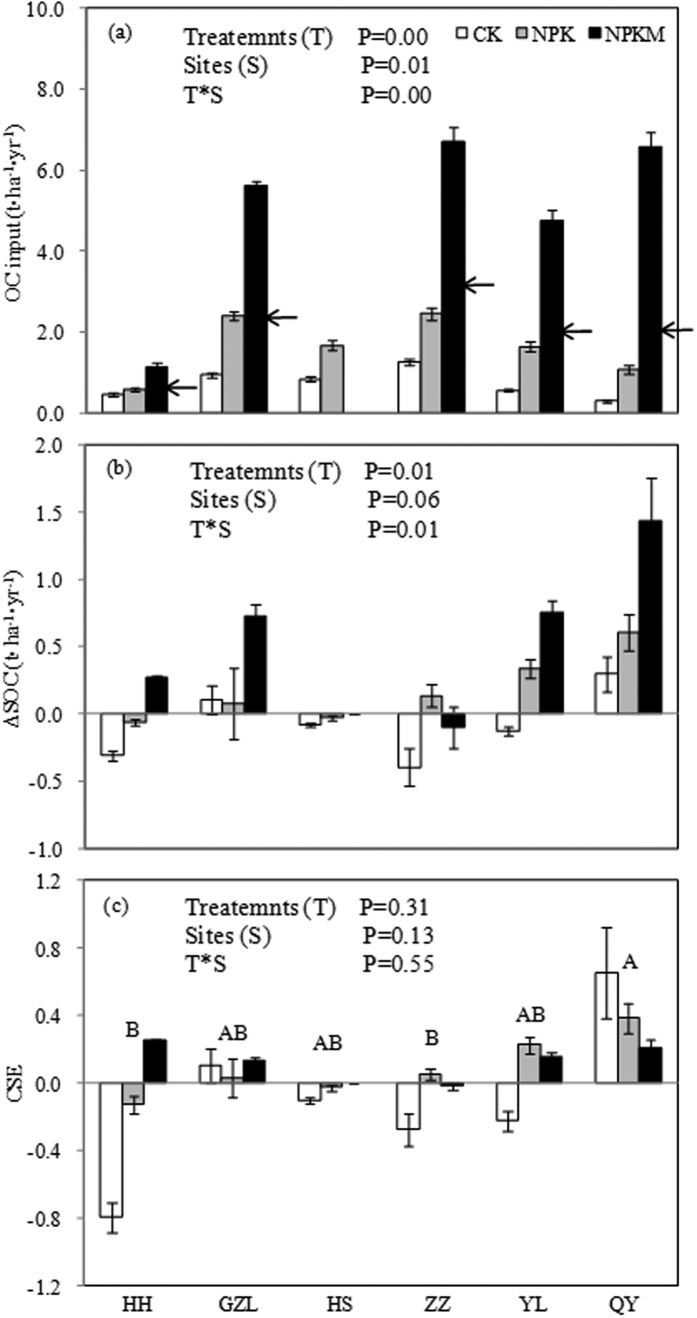
The main and interactive effects of fertilization treatment (T) and site (S) on C input, change in SOC storage (ΔSOC) and CSE at the six fertilization experimental sites in China based on repeated measure two-way ANOVA. The different capitalized letters denote significant difference between sites at P < 0.1. In panel (a), bar height below each arrow denotes the yearly mean OC input via crop residual at each site and the rest from manure input.

The annual C inputs (1979~2013) range from 0.2 to 1.8 t C ha^−1^yr^−1^ under CK, 0.3 to 3.5t C ha^−1^yr^−1^ under NPK, and 0.4 to 10.0 t C ha^−1^ under NPKM, with C input generally higher in ZZ and GZL and lower in HH (Fig. 1a–c). Two-way ANOVA showed that there are significant interactive effect of fertilization and site on OC input (*p*-value < 0.01, Fig. 2a). A post hoc test showed that there are significant fertilization effects on C input in GZL (NPKM > NPK > CK), and there are also significant fertilization effects on C input in HH, ZZ, YL and QY (NPKM > NPK, CK).

### Change of SOC under long-term fertilization

The variation of ∆SOC was more pronounced during 1979–1995 than during 1996–2013 (Fig. 1d–f). On average, the ∆SOC decreased at QY and increased at GZL under all fertilization treatments during the entire experiment duration. Two-way ANOVA showed significant interaction of fertilization and site on ∆SOC (*p*-value = 0.01, Fig. 2b). The post hoc test showed that there were significant fertilization effects on ∆SOC in HH (NPKM > NPK > CK), and there are also significant fertilization effect on ∆SOC in YL (NPKM > CK).

### CSE under long-term fertilization

The variation of CSE showed a similar pattern to that for ∆SOC, i.e. there is much greater variations during 1979–1995 than 1996–2013 (Fig. 1d–f). In most sites, CSE under NPKM treatment appeared to be more than zero, which is larger than that for CK or NPK treatments at the same site. Two-way ANOVA showed marginally significant site effect on CSE (*p*-value = 0.13) and the post hoc test showed CSE is lower at HH and ZZ than QY (Fig. 2c).

### The proportional contributions to variations in CSE

**Figure 3:**
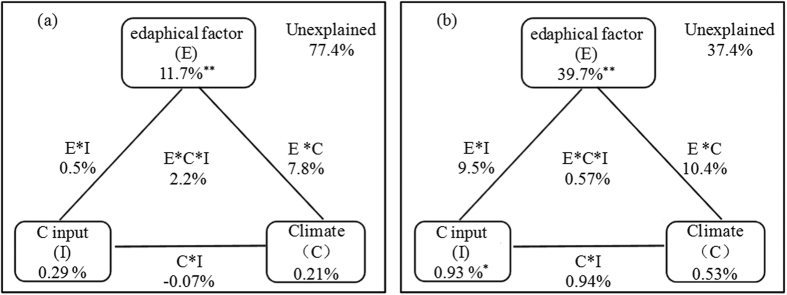
The proportional contributions (%)of edaphic factor (E), climate (C), C input (I) and their interactions on variance of CSE at the six fertilization experimental sites in China based on VPA method conducted among (**a**) all fertilization treatments and sites and (**b**) only at Zhengzhou (ZZ).

Among all fertilization and site treatments, 22.6% of total variance was explained by all factors (*p*-value < 0.01), while 11.7%, 0.3% and 0.2% can be explained by edaphic factor, C input and climate, respectively (Table 1, Fig. 3a). The amount of variance explained by interactive terms of edaphic, C input and climate in CSE were less than that by edaphic factor (Fig. 3a,b). In ZZ, 62.6% of total variance was explained by all factors (p-value < 0.01), while 39.7% of the variance can be explained by edaphic factor alone (Table 1, Fig. 3b).

Under each fertilization treatment or site, the total variance that can be explained are 49.1%(CK), 40.5% (NPK), 28.9% (NPKM), 62.6% (ZZ), 41.2% (HH), 37.2% (GZL), 36.5% (YL), 24.1% (QY), and 15.9% (HS) (Table 1). The proportional variances that can be explained by edaphic factor are 25.2% (CK), 19.4% (NPK) and 8.9% (NPKM), or 39.7% (ZZ), 29.9% (GZL), 20.5% (HH), 15.0% (QY), 13.2% (YL) and 10.6% (HS), respectively. The proportional variances that can be explained by C input (<1.0%) or climate (<1.4%) are substantially smaller than that explained by edaphic factor (Table 1). The amount of total variance explained by edaphic factor followed a descending order (CK > NPK > NPKM) while that explained by C input coincided with the pattern of C input due to fertilizations (CK < NPK < NPKM).

Each individual edaphic factor that contributed most to the variance of CSE differ among fertilization or site treatments. These factors identified include AN (all treatments, CK, NPK, HH, HS), pH (NPKM, HH, YL, QY), BD (HH, GZL) and TK (HH, ZZ), which explained 7.9%, 4.7%, 10.2%, 7.0%, 3.5%, 1.3%, 5.6%, 6.2%, 2.6%, 17.5%, 4.6% and 13.4%, respectively (Table 1).

## Discussions

### The variance of CSE is primarily affected by edaphic factor across sites

SOC storage has been identified to be closely related with MAT and MAP over large geographical scale^37^,^38^, and were slightly enhanced by soil amendments (e.g. farm manure, straw or other crop tissue) at agricultural zones in China and India^19^,^39^. Through VPA method, this study however explicitly identified the edaphic factor the most important driver on variance of CSE (i.e. 8.9–39.7%) under individual fertilization treatment, site or across all fertilization treatments and sites. The observed pattern can be addressed in the following three aspects.

**Figure 4:**
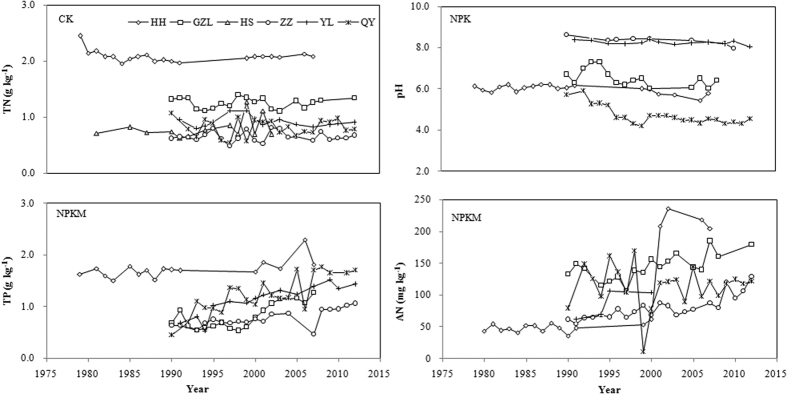
The temporal trends of soil properties in three treatments from 1979–2013 at the six fertilization experimental sites in China.

First, the six soil types included dark brown soil, black soil, fluvo-aquic soil, losses soil, and red soil which are developed from four distinct parent materials including LuvicPhaeozems, Calcariccambisoil, Cumulicanthrosol and Eutriccambisol, respectively (Table 2). These materials differ in mineral type, composition and nutrient concentrations^40^, which can lead to very different soil physiochemical characteristics. At the initiation of these long-term experiments, soil physical and chemical properties indeed showed high variations such as BD (1.10~1.58 g∙cm^−3^), pH (5.7~8.6), AN (50~131 mg∙kg^−1^), and AK (50~194 mg∙kg^−1^) (Table 1). After more than two-decade intensive fertilization, key soil properties may stayed relatively constant or changed to certain extent under the same treatment in each site, but the differences in these properties between sites remained consistently large (Fig. 4). On the other hand, long-term fixed soil experiments are important platforms to monitor soil change over decades or centuries^41^,^42^. To incorporate the diverse soil and cropping systems, the LTNSFFEMN specifically selected more than ten typical agricultural soils with distinct soil and cropping systems^36^.

Second, in spite of varying climate conditions across sites (i.e. −1.5~18.5 °C in MAT, 510~1255 mm in MAP), the number of only six sites selected in this study is much fewer than those large scale studies conducted across a large number of sites. Based on a study at twenty-two sites across a large gradient of MAT (i.e. −6 °C to 24 °C), even very different soil types were included located in various ecosystem types (i.e. arable, grassland, forest), the influence of soil factors were low. The number of studies thus likely minimized the probability of detecting the influence of soil^43^. Increasing evidence supported that edaphic characteristics were usually less likely to be detected when a large number of sites were synthesized together^38^,^43^,^44^. It is climate conditions, not soil factor that may play a more important role in regulating SOC dynamics over large geographical regions.

Third, edaphic characteristics explained 53.3% of the variation in soil microbial communities structure based on 18 sites (i.e. 1 ha^−1^ of each plot) under three fertilization treatments, and the amount of variance explained exceeds the amount of variance explained by climate and managementfactors^45^. Soil microbes play a key role in regulating SOC decomposition and formation^46^,^47^ resulting in SOC change. This suggests the above findings are supportive to our current results. On the other hand, the relatively lower percent of variance explained by edaphic factor in our study (8.9–39.7%) can be due to other abiotic processes leading to SOC additions or losses such as manure amendments, plant residual and root exudates and DOC leaching^18^,^48^.

### The major edaphic factors responsible for variance of CSE varied between different sites

First, among individual soil factors, soil available N can explain 7.9% of total variation of CSE in six sites corroborating that it is the most important limiting factor in these typical upland soils in China. It has been long known that nitrogen is a key to the ecosystem function, and the available nitrogen content was used frequently to index plant nutrient status, i.e. the balance of plant demand and soil supply of nutrients in croplands^49^,^50^,^51^. Specifically, this study showed that available N was identified to be the main factor on CSE variance under CK and NPK treatments. For the CK treatment, there is no fertilizer input for more than twenty years, and N supply depends solely upon basic soil fertility, while nitrogen has been always a limiting factor on crop growth and belowground C transformations in such a infertile soil^52^,^53^,^54^,^55^; For the NPK treatment, the large amount of N fertilizer in addition to P and K inputs in all these sites enhanced crop yields by 140–680%^54^,^56^,^57^ and via the increased plant and root C inputs elevated soil microbial activity, microbial biomass and SOC accumulation^18^,^58^,^59^. At the HH site, available N is the most important factor on explaining variance of CSE, likely associated with lower annual N fertilizer input than other sites. These positive or negative effects of N fertilizer input on crop C inputs to soils represent an important mechanism via soil available nitrogen on the CSE under these fertilization treatments without external manure inputs. For the NPKM treatment, it is not soil available nitrogen but pH that was identified to be the most important factor for explaining the CSE variance suggesting that as compared with NPK treatment, manure inputs in NPKM treatment most likely diminished the nitrogen constrains on crop growth and subsequent influence on SOC turnover^60^,^61^.

Second, pH was identified to be the only significant edaphic factor explaining CSE variance at QY and YL, and the most important one in the NPKM treatment; pH was also one of the significant factors explaining CSE at HH. This is likely due to effects of pH on the turnover and accumulation of organic matter. For example, a study showed that pH is significantly negatively correlated with SOM content and this relationship is true for sites at the Northwestern China^62^ such as YL included in our study. At the originally acidic site such as QY, crop systems with long-term fertilization (i.e. NPK) or no fertilization were subjected to acidification which lowered crop yields significantly^63^,^64^,^65^ and subsequently led to lower C inputs indirectly^18^. On the other hand, low pH in acidic soils can reduce dissolved organic carbon^66^ and alter the interactions of SOM-mineral in tropical soil^67^, which collectively can lead to changes in SOC.

Third, BD was identified as the most important edaphic factor explaining variance of CSE in GZL and it was also one of the most important edaphic factors at the most northern site (i.e. HH). This result is consistent with that found in a clayey soil of Tunisia using a similar method to VPA^68^. A study using VPA showed that BD was identified to be the main driving factor on the variation in microbial communities^45^, which indicates that BD can contribute to explanation of CSE via the key role of microbes on soil carbon decomposition and formation^69^,^70^,^71^. Specifically, the amount of variance in CSE that can be explained by BD is much higher at GZL than at other sites, most likely due to the soil sampling conducted along the ridging made to promote maize growth at GZL^72^,^73^, which method differed substantially from the general soil sampling at other sites.

### The relative importance of C input and climate on variance of CSE in upland of China

The proportional contribution of C input and climate to the total variance of CSE are 0.3~7.9% and 0.2~3.1%, respectively, which are less than that explained by edaphic factor, i.e. 8.9–39.7%. In spite of less important role of C input to variance of CSE than edaphic factor, the increasing amount of C input due to fertilizations (CK < NPK < NPKM) lessened the relative contribution of edaphic factor (*p*-value < 0.05) but enhanced the relative contribution of C input to total variance of CSE. In particular, we found a highly significant percentage contribution of manure to CSE variance at GZL (4.9%, *p*-value < 0.05), suggesting the increasing importance of manure input in regulating SOC sequestration although it does not change the dominant impact exerted by soil on CSE (29.9%, *p*-value < 0.01). It remains largely unknown how manure amendments have been transformed and deposited into different SOM pools mechanistically. It is likely that specific organic C components of manure material may be preferentially decomposed and incorporated into different SOC pools. From this perspective, this finding echoed with a recent study showing that long-term use of manure enhanced microbial routing of specific mono saccharides into different particular organic matter fractions, thus maintaining SOC content over decades^74^.

The proportional contribution of climatic factor on CSE variation does not reach a significant level suggesting MAT and MAP had little effects on CSE variance across the selected sites. Although air temperature and precipitation were identified as the significant drivers of SOC change over regional to global scales^22^,^23^,^38^,^43^,^75^, the current six sites selected from typical croplands in China have been preferentially established in distinct soil types, which potentially tended to detect mechanistic drivers at the plot level not the large scale. On the other hand, favorable temperature and precipitation may directly affect aboveground plant growth^27^, but their effects on belowground root and microbial processes can be largely mediated by the physical constrains in soil resulting in varying patterns of soil temperature and moisture conditions^76^,^77^. Furthermore, several other studies concluded that there has been no consistent climatic driver identified for SOC changes in the research plots in Palace Leas and Park Grass of UK^78^,^79^. A plot level field study showed that climate only accounted for 0.14% in mass remaining in a straw decomposition experiment^28^.

## Conclusions

Long-term chemical fertilizers alone or combined with manure input significantly increased soil organic carbon stock but the rate of SOC change per unit C input (i.e. CSE) varied substantially across sites. The study identified that edaphic factor can explain much larger amount of variance in CSE (i.e. up to 39.7%) than climate and management (i.e. C input) suggesting that soil properties play a major role during SOC turnover and accumulation across the selected Chinese cropland soils. Furthermore, available N content, pH, BD and TK were frequently identified to explain larger amount of variance in CSE than other edaphic variables suggesting SOC sequestration is likely driven by different soil property via varying mechanistic soil processes. This study demonstrated differential mechanisms in control of decade-long SOC transformations over large geographical regions. Overall, this study informed the need to develop different soil management plan in order to promote soil carbon sequestration under long-term fertilization. To expand the current analysis, future studies should account for more potentially available soil, climatic and management variables (i.e. soil temperature, soil moisture, irrigation) across more experimental sites in China.

## Materials and Methods

### Site characteristics, soil collection and fertilization experiment

**Figure 5:**
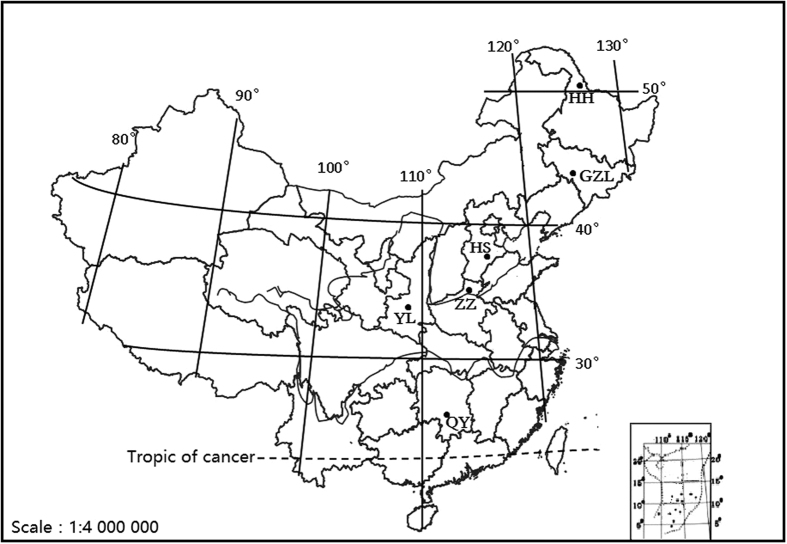
Locations of long-term fertilization experiments at six typical croplands in China. These sites located from north to south include Heihe (HH) in Heilongjiang province, Gongzhuling (GZL)in Jilin province, Hengshui (HS)in Hebei province, Zhengzhou (ZZ)in Henan province, Yanglin (YL)in Shanxi province, and Qiyang (QY) in Hunan province. The map was downloaded from Chinese National Basic Geographical Information System 1:4000000 database (http://www.cehui8.com/3S/GIS/20130702/205.html) and is edited using ArcGIS 9.3 (http://www.esri.com/).

In order to evaluate fertilizer efficiency, the Long-term National Soil Fertility and Fertilizer Efficiency Monitoring Network (LTNSFFEMN) was established in more than ten typical agricultural areas of China in the late 1980s^34^. Soil types include dark brown soil, black soil, loamy soil, loess soil, red soil. We chose six of these long-term experiments with different soil types and cropping systems along a latitudinal transect of China (Fig. 5). These sites located from north to south are Heihe (HH) in Heilongjiang province, Gongzhuling (GZL) in Jilin province, Hengshui (HS) in Hebei province, Zhengzhou (ZZ) in Henan Province, Yanglin (YL) in Shanxi province, and Qiyang (QY) in Hunan province (Fig. 5). The six sites differ in mean annual temperature (up to >20 °C difference), mean annual precipitation (MAP) (about 3 times difference) and are located in distinct climatic zones (Table 3). These sites also vary in the cropping system. HH and GZL are the two sites under mono-cropping cultivation of wheat or maize, and HS, YL, ZZ, QY are the four sites under double-cropping cultivation (Table 3). The annual soil sample collections were conducted in September or October after crop harvest at all sites. A composite soil sample was annually obtained by homogenizing three soil samples in the field. Annual crop yields of wheat and maize were recorded.

The fertilization experiments were initiated in 1979 at HH, 1981 at HS and 1990 at other sites. The plot areas differ between sites, i.e. 7.5 m × 5 m in HS, 20 m × 10.6 m in HH, 10 m × 57 m in GZL, 8.6 m × 5 m in ZZ, 14 m × 14 m in YL, and 14 m × 14 m in QY. This study collected datasets that specifically focused on three treatments: cropping but no fertilizer input (CK), chemical nitrogen, phosphorus and potassium fertilizers (NPK), and NPK with animal manure (NPKM). For the chemical fertilization treatments, urea was applied as chemical N fertilizer in six sites. Calcium superphosphate was applied as P fertilizer in HS, ZZ, YL and QY, and diammonium phosphate was applied as P fertilizer in HH and GZL. Potassium chloride was applied as K fertilizer in GZL, ZZ and QY, and potassium sulphate was applied as K fertilizer at YL.

For the NPKM treatment, 30–40% of total nitrogen was applied as chemical fertilizer and the rest derived from the animal or crop derived manure. These include horse manure in HH, pig manure in GZL and QY, cow manure in ZZ and YL, and plant residues in six sites. The organic C content is 361 g kg^−1^ in horse manure, 398 g kg^−1^ in pig manure in GZL, 414 g kg^−1^ in pig manure in QY, 368 g kg^−1^ cow manure in ZZ and 310 g kg^−1^ in cow manure in YL. Horse manure was applied once every three years in HH, pig manure was applied each year before seeding in GZL, cow manure was applied as basal in every autumn before wheat planting in ZZ and YL, and pig manure was applied once as base fertilizer before seeding in QY. One third of N fertilizer was applied as base fertilizer before seeding and the rest as topdressing at the jointing stage. All the P and K fertilizers were applied as base fertilizers before seeding. In the NPKM treatment, the certain amount of manure was applied to provide the same amount of nitrogen as chemical N fertilizers. The input rate of chemical fertilizers and manure in each site were listed in Table 4. To note, the manure input is more than twice at QY relative to that in other sites.

Given the local climate and convention, other agricultural management practices besides fertilization also vary between sites. Tillage is a common practice in all six sites but differs in intensity, timing, depth and means. There are three different tillage means including rotary tillage (HS and YL, one time before wheat planting), tractor plow harrow (ZZ, twice before maize planting before 2009 then none after 2009) and general plow (HH, GZL and QY, one time shortly after crop harvest). The depth of tillage is 15–20 cm in all sites except a 30-cm depth in ZZ. Irrigation and pest control were also employed in each site and could vary substantially due to the amount of rain, the crop varieties, the pesticide types and local conditions. Even within each long-term experiment, its sustainability could be challenged by the natural and human resources (Li and Xu, 2015)^80^. In spite of these potential management uncertainties, we assume that the overall effect varies little between sites. As long as there are the long-term continuous and accurate records of the key fertilizers and manure inputs, they will allow us to derive external C input and carbon sequestration efficiency in our current study.

### Physiochemical analysis

Prior to analysis, soil samples were air-dried and passed through a 2-mm sieve. Crop residues, root material and gravels were identified and removed from the composite soil sample before they were subject to physiochemical analysis. Separate soil samples were collected to determine the bulk density at each soil depth^81^. Soil samples for SOC measurements were pretreated with 0.5 M HCl to remove carbonates^82^ and then ball-milled. The concentration of TOC and TN were determined by CHN analyzer (EA-3000, EuroEA Elemental Analyzer, EUROVECOR S P A, ITALY). Available and total concentration of nutrients (N, P, K) were quantified based on classical analytical methods^83^,^84^. The clay content, texture, soil pH (1:1 v/v water) and bulk density were also measured by classical analytical methods^78^. Soil type at each site was classified based on United Nations Food Agriculture Organization (FAO) soil taxonomy system. The relevant edaphic factors were presented in Table 2. The MAT and MAP are the mean value of every day. Temperature and precipitation data for each site were collected from the nearest meteorological station of the China Meteorological Administration. Organic carbon input into topsoil included root system, and addition of organic manure. The annual rates of carbon input by roots in maize and wheat were estimated as 30% of the above-ground carbon biomass^85^,^86^.

### Soil carbon sequestration efficiency (CSE)

The soil carbon sequestration efficiency (CSE) is derived using the following equation:


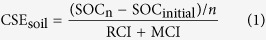


SOC_n_ and SOC_initial_ is the SOC storage in n’th year (t C∙ha^−1^); n is experiment duration (year). RCI is the mean annual OC input of crop stubble of wheat or corn to soil per year (t C∙ha^−1^); MCI is mean annual OC input from manure (t C∙ha^−1^).


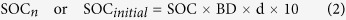


SOC is the OC content on topsoil in n’th or initial year (g∙kg^−1^); BD is the soil bulk density (g∙cm^−3^); d is the soil depth (0.2 m); 10 is the conversion factor.


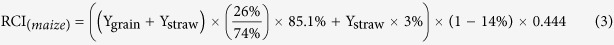



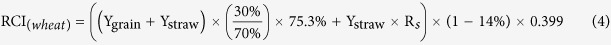


Y_grain_ and Y_straw_ is the grain and straw yield of the wheat/maize (kg∙ha^−1^), respectively; 26%/74% and 30%/70% are the allocation ratio of C in above-ground and below-ground for maize and wheat, respectively; 85.1% and 75.3% are the percentile of root biomass of maize and wheat in 0–20 cm soil, respectively; 3% is the ratio of maize stubble on straw biomass; R_s_ is the ratio of wheat stubble on straw biomass, but it is different in treatments; 14% is the average moisture content of wheat and maize samples; 0.444 and 0.399 are the proportion of organic carbon content of dried maize and wheat.


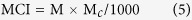


M is the annual manure inputs (kg∙ha^−1^), M_*c*_ is the C content of manure (g∙kg^−1^), and 1000 is the conversion factor.

### Statistical analysis

The repeated measured two-way ANOVA was used to test the effects of fertilization treatments (CK, NPK, NPKM), sites (HH, GZL, HS, ZZ, YL, QY) and their interaction on OC input, SOC storage change (ΔSOC) and CSE based on collections from the initial year to 2012. All analyses were conducted using SPSS (SPSS Statistics 20). The significance level is set at P < 0.05.

Variance partitioning analysis (VPA) method is used to obtain the quantitative contributions of edaphic, climatic and C input as well as their interactions to CSE. To conduct the VPA procedure in this study, the CSE of per year is the dependent variable, the edaphic factor, climate, and C input are the independent variable. The edaphic properties included total nitrogen (TN), phosphorus (TP), potassium (TK), available N (AN), P (AP), K (AK), pH, soil bulk density (BD), soil sand, soil silt particle content, soil clay content of each year from the initial year to 2009 or 2012 under each fertilization treatment at six sites. OC input includes stubble carbon input (SC), manure input, annual mean OC input (AC), total OC input (TC) from the initial year to 2009 or 2012 under each fertilization treatment at six sites. Climatic factors included mean annual temperature (MAT) and mean annual precipitation (MAP) for each fertilization treatment at six sites. Taking into account the collinearity, indicators that are not significantly correlated were selected for running VPA. The analysis aims to obtain the contributions of each factor (i.e. edaphic, C input and climate), the interactions among three factors, and each individual variable within each factor and their interactions among these variables. The significant level is set at P < 0.05. All analyses were conducted using R program (R version 3.2.2)^87^.

## Additional Information

**How to cite this article**: Liang, F. *et al*. Three-decade long fertilization-induced soil organic carbon sequestration depends on edaphic characteristics in six typical croplands. *Sci. Rep.* **6**, 30350; doi: 10.1038/srep30350 (2016).

## Figures and Tables

**Table 1:** The proportional contribution (%) of individual and total edaphic factor, climate, and C input on variance of CSE based on VPA method in long-term fertilization experiments at six typical croplands in China.

Category	Indictors	Overall	Treatments			Sites					
			CK	NPK	NPKM	HH	GZL	HS	ZZ	YL	QY
Edaphic factor	TN	1.4^**^	0.0	0.5	0.0	0.4	0.9	1.6	0.1	0.2	0.1
	AN	7.9^**^	4.7^**^	10.2^**^	1.1	7.0^**^	3.4	3.5	2.4	0.2	1.3
	TP	3.0^**^	3.0^*^	4.8^**^	0.0	2.1	2.6	1.7	2.7^*^	0.0	0.0
	AP	0.4	0.5	0.6	0.0	0.1	0.4	0.8	2.1	0.8	0.0
	TK	0.6	1.2	0.0	0.4	4.6^**^	0.4	0.0	13.4^**^	0.8	0.1
	AK	0.1	0.0	0.0	0.2	0.1	0.0	2.8	0.1	1.4	1.6
	pH	0.0	1.2	0.0	1.3	1.3	0.0	0.0	0.5	5.6^*^	6.2^*^
	BD	0.3	0.0	1.3	0.2	2.6^*^	17.5^**^	0.6	1.7	2.6	1.2
	Total^a^	11.7^**^	25.2^**^	19.4^**^	8.9	20.5^**^	29.9^**^	10.6	39.7^**^	13.2	15.0
C input	SC	0.1	0.0	0.2	0.7	0.9	0.7	4.6	0.4	6.8^*^	0.7
	MC	0.0	–	–	0.0	0.1	4.9^*^	–	0.0	0.5	4.3
	TC	0.1	0.4	0.7	0.0	0.0	0.5	5.8	0.6	2.0	0.7
	Total^a^	0.3	0.4	0.7	1.0	0.9	7.9	7.8	0.9	7.1	5.8
Climate	MAP	0.2	0.0	0.0	0.0	0.9	0.2	0.7	0.4	2.6	2.0
	MAT	0.1	0.4	1.0	0.8	1.2	2.3	0.9	0.3	0.2	0.9
	Total^a^	0.2	0.5	1.4	0.8	1.9	2.4	1.3	0.5	3.1	2.4
Total ^b^	–	22.6^**^	49.1^**^	40.5^**^	28.9^**^	41.2^**^	37.2^*^	15.9	62.6^**^	36.5^**^	24.1

Abbreviations: SC: stubble carbon input; MC: manure C input; TC: total OC input as the sum of SC plus MC. Other abbreviations were presented in Table 2. *P < 0.05, ^**^P < 0.01.

^a^Represents total variance explained by single or interactive edaphic factor; ^b^represents total variance explained by single or interactive edaphic, C input and climate factor.

**Table 2:** Soil characteristics (0~20 cm) at the initiation of long-term fertilization experiments at six typical croplands in China.

Site	SOC g∙kg^−1^	TN g·kg^−1^	C:N	AN mg·kg^−1^	TP g·kg^−1^	Olsen-P mg·kg^−1^	TK g·kg^−1^	AK mg·kg^−1^	pH	BD g∙cm^−3^	Clay %	Soil type^a^	Soil type^b^
HH	26.5	2.5	10.6	50	1.7	7.8	2.7	50	6.1	1.37	33	Dark brown soil	LuvicPhaeozems
GZL	13	1.42	9.2	131	1.5	23	24.6	160	7.2	1.19	32	Black soil	LuvicPhaeozems
HS	6.7	1.0	6.7	51	0.6	12	18	113	8.4	1.58	23	Fluvo-aquic soil	Calcariccambisoil
ZZ	6.7	0.67	10	51	0.6	6.5	16.9	74	8.3	1.24	13	Fluvo-aquic soil	Calcariccambisoil
YL	7.4	0.8	9.3	63	0.6	9.6	21.6	194	8.6	1.41	21	Lou soil	Cumulicanthrosol
QY	8.6	1.07	8.0	79	0.5	11	13.3	122	5.7	1.10	41	Red soil	Eutriccambisol

SOC: soil organic carbon; TN: total nitrogen; AN: available nitrogen; TP: total phosphorus; TK: total potassium; AK: available potassium. BD: bulk density; Clay: clay content; SP: soil porosity.

^a^Based on China soil taxonomy.

^b^Based on United Nations FAO soil taxonomy.

**Table 3:** Site characteristics of long-term fertilization experiments at six typical croplands in China.

Site	Coordinate	Altitude (m)	MAT (°C)	EAT (°C)	MAP	MAE	Climatic zone in China	Cropping system	Experiment Initiation
HH	50°15'11″N 127°27'07″E	180	−1.5	2180	510	650	Mild-Temperate, Semi-Humid	Mono-cropping Wheat or Soybean	1979
GZL	43°30'23″N 124°48'34″E	220	4.5	1700	589	1400	Mild-Temperate, Semi-Humid	Mono-cropping Maize	1990
HS	37°44'00″N 115°47'00″E	28	13.0	4889	550	1300	Warm-Temperate, Semi-Humid	Double-cropping Wheat-maize	1981
ZZ	35°50'00″N 113°42'00″E	59	14.5	2661	615	1450	Warm-Temperate, Semi-Humid	Double-cropping Wheat-maize	1990
YL	34°17'51″N 108°00'48″E	524	13.0	2323	575	993	Warm-Temperate, Semi-Humid	Double-cropping Wheat-maize	1990
QY	26°45'00″N 111°52'00″E	120	18.5	3429	1255	1470	Sub-Tropical, Humid	Double-cropping Wheat-maize	1990

HH: Heihe in Heilongjiang province; GZL: Gongzhuling in Jilin province; HS: Hengshui in Hebei province;

ZZ: Zhengzhou in Henan province; YL: Yanglin in Shanxi province; QY: Qiyang in Henan province.

MAT: mean annual temperature; EAT: effective annual temperature;

MAP: mean annual precipitation; MAE: mean annual evaporation.

**Table 4:** Input rates of chemical fertilizers (kg ha^−1^) and manure (t ha^−1^) in long-term fertilization experiments at six typical croplands in China.

Site	Fertilizer	CK		NPK		NPKM	
		Wheat	Maize	Wheat	Maize	Wheat	Maize
HH	N-P-K	0-0-0^a^	NA	38-17-0	NA	38-17-0	NA
	Manure	0		0		15	
GZL	N-P-K	0-0-0	NA	165-36-69	NA	50-36-69	NA
	Manure	0		0		23	
HS	N-P-K	0-0-0	0-0-0	45-26-0	45-0-0	NA	NA
	Manure	0	0	0	0		
ZZ	N-P-K	0-0-0	0-0-0	165-36-68	188-41-78	50-36-68	188-41-78
	Manure	0	0	0	0	15^b^	0
YL	N-P-K	0-0-0	0-0-0	165-58-68	188-25-78	165-58-68	188-25-78
	Manure	0	0	0	0	13	0
QY	N-P-K	0-0-0	0-0-0	90-16-30	210-37-70	27-16-30	63-37-70
	Manure	0	0	0	0	10~15^c^	25~35

NA: no crop.

^a^Denotes fertilization rates are zero for all three fertilizers;

^b^Is calculated based on C/N (i.e. 25) and carbon content (i.e. 19.8%) in cow manure;

^c^Denotes a range.
